# A Review on Disorder-Driven Metal–Insulator Transition in Crystalline Vacancy-Rich GeSbTe Phase-Change Materials

**DOI:** 10.3390/ma10080862

**Published:** 2017-07-27

**Authors:** Jiang-Jing Wang, Ya-Zhi Xu, Riccardo Mazzarello, Matthias Wuttig, Wei Zhang

**Affiliations:** 1 Center for Advancing Materials Performance from the Nanoscale, State Key Laboratory for Mechanical Behavior of Materials, Xi’an Jiaotong University, Xi’an 710049, China; wangjiangjing@stu.xjtu.edu.cn (J.-J.W.); yazhi_xu@stu.xjtu.edu.cn (Y.-Z.X.); 2 Institute for Theoretical Solid-State Physics, JARA-FIT and JARA-HPC, RWTH Aachen University, 52074 Aachen, Germany; mazzarello@physik.rwth-aachen.de; 3 Institute of Physics IA, JARA-FIT and JARA-HPC, RWTH Aachen University, 52074 Aachen, Germany; wuttig@physik.rwth-aachen.de

**Keywords:** metal–insulator transition, disorder, Anderson insulator, electron localization, phase-change materials

## Abstract

Metal–insulator transition (MIT) is one of the most essential topics in condensed matter physics and materials science. The accompanied drastic change in electrical resistance can be exploited in electronic devices, such as data storage and memory technology. It is generally accepted that the underlying mechanism of most MITs is an interplay of electron correlation effects (Mott type) and disorder effects (Anderson type), and to disentangle the two effects is difficult. Recent progress on the crystalline Ge_1_Sb_2_Te_4_ (GST) compound provides compelling evidence for a disorder-driven MIT. In this work, we discuss the presence of strong disorder in GST, and elucidate its effects on electron localization and transport properties. We also show how the degree of disorder in GST can be reduced via thermal annealing, triggering a disorder-driven metal–insulator transition. The resistance switching by disorder tuning in crystalline GST may enable novel multilevel data storage devices.

## 1. Introduction

Solids can be categorized into metals and insulators according to their electrical resistivity at zero temperature, i.e., metals have finite resistivity, while that of insulators diverges. According to the conventional band theory, insulating or metallic behavior depends on whether the energy bands of the solid are fully or partially filled [1]. However, many transition-metal oxides (such as NiO) with a partially filled *d*-band were found to be good insulators [2]. N.F. Mott explained this phenomenon by including interactions between electrons, whereby the valence electrons in the partially filled *d* band are forced to stay on the lattice sites due to the strong Coulomb interactions [3,4,5]. By doping or applying pressure, a metal–insulator transition (MIT) can occur in Mott systems. A different mechanism for electronically driven MITs was proposed by P. W. Anderson, who emphasized the role of disorder in inducing electron localization [6,7,8]. Although the two concepts are rather different, in many systems, it is difficult to disentangle the effects of electron correlation and disorder. For example, in doped semiconductors, such as phosphorus doped silicon [9,10,11], correlation effects are important as the increasing charge carrier density reduces the ratio between electron-correlation and Fermi energy, triggering an insulator-to-metal transition. However, the effects of disorder cannot be neglected, because the dopants are arranged randomly on the lattice sites of silicon. Recently, compelling evidence for purely disorder-driven MIT has been obtained in crystalline phase change materials (PCMs) from both experiments [12,13,14] and simulations [15,16,17]. In this work, we will give a comprehensive review of this active research area.

Phase change materials (PCMs) are a special group of chalcogenides with a peculiar combination of physical properties that have a variety of important applications in non-volatile electronic, optical and photonic data storage devices [18,19,20,21,22,23], memristors and processors [24,25,26,27,28], spintronics [29,30,31,32,33], display and data visualization applications [34], and so on. PCMs can undergo rapid and reversible phase transitions between an amorphous and a crystalline phase upon heating. The significant difference in electrical and optical properties allows to encode digital information [18,35,36]. The most widely used and studied phase change materials are GeSbTe compounds along the pseudo-binary GeTe-Sb_2_Te_3_ line [37,38,39]. Electron–electron correlation effects are shown to be weak in PCMs owing to the special bonding mechanism, i.e., resonant bonding, which results in high dielectric constants [40,41]. On the other hand, a significant amount of atomic disorder is present in GeSbTe compounds [38,42], and plays an essential role in shaping their electronic properties. By tuning the disorder in crystalline GeSbTe compounds, the electrical resistance can vary by three orders of magnitude at room temperature. Combined with another three orders of magnitude resistance difference with respect to the amorphous phase, the resistance window is sufficiently large to enable multi-level data storage, which could lead to a drastic increase in data storage density.

## 2. Discussions

### 2.1. Atomic Disorder in GST Crystals

**Figure 1:**
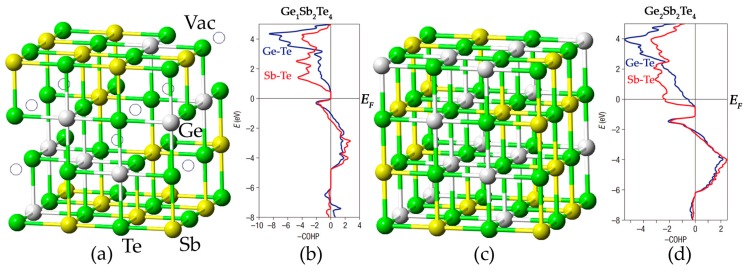
Cubic rocksalt structures of (**a**) Ge_1_Sb_2_Te_4_ and (**c**) Ge_2_Sb_2_Te_4_. Atomic vacancies, Ge, Sb and Te atoms are rendered with hollow circles, grey, yellow and green spheres, respectively. The crystal orbital Hamilton population (COHP) bonding analyses of Ge_1_Sb_2_Te_4_ and Ge_2_Sb_2_Te_4_ are shown in (**b**,**d**), where the negative values (of –COHP) represent antibonding interactions (left side), while positive values represent bonding ones (right side). The Ge–Te and Sb–Te contacts are shown in blue and red, respectively. Adapted with permission from Reference [45] © 2007 Nature Publishing Group.

GeSbTe alloys form a special cubic rocksalt structure upon rapid crystallization from the amorphous phase, in which the anion-like sublattice is taken by Te atoms, while the cation-like sublattice is occupied by Ge, Sb atoms and atomic vacancies in a random fashion [38,43,44]. Although this phase is metastable, it can be robust at room temperature for decades, and plays an important role in phase change memory devices. Stoichiometric GST compounds locate in the GeTe-Sb_2_Te_3_ pseudo-binary line, i.e., (GeTe)*_m_*(Sb_2_Te_3_)*_n_*, and the most studied and used compounds include Ge_1_Sb_2_Te_4_ (*m* = 1, *n* = 1), Ge_2_Sb_2_Te_5_ (*m* = 2, *n* = 1), Ge_8_Sb_2_Te_11_ (*m* = 8, *n* = 1). In Figure 1a, Ge_1_Sb_2_Te_4_ is shown (we denote Ge_1_Sb_2_Te_4_ as GST starting from now on). Ge, Sb and Te atoms are rendered with grey, yellow and green spheres, while atomic vacancies are rendered with hollow circles. Clearly, the concentration of atomic vacancies is huge, e.g., 12.5% for Ge_1_Sb_2_Te_4_, which is several orders of magnitude larger than that in other semiconductors, such as silicon.

Why such huge amount of vacancies can be present in GST crystals? This question was answered by Density Functional Theory (DFT) simulations [46,47,48] and quantum chemistry bonding analysis [49]. In Ref. [45], a hypothetical rocksalt Ge_2_Sb_2_Te_4_ alloy was modeled, as shown in Figure 1c. The formation energy of atomic vacancies is negative, ~−0.5 eV, indicating that the system is stabilized upon forming vacancies. This is in striking difference with silicon, where the formation of vacancies is quite unfavorable, 3.3 eV [50]. The crystal orbital Hamilton population (COHP) bonding analysis [49,51,52,53] provides a deeper understanding of this unconventional behavior in GST crystals. This method dissects the electronic structure into bonding and antibonding interactions. A high antibonding contribution at the Fermi level *E_F_* indicates that the system has chemical instability. In certain cases, such unfavorable interaction would result in a complete collapse of the crystal lattice [54,55]. The comparison between rocksalt Ge_1_Sb_2_Te_4_ and Ge_2_Sb_2_Te_4_ is shown in Figure 1b,d. Significant antibonding interactions (–COHP < 0) are present at the *E_F_* of Ge_2_Sb_2_Te_4_, which are completely removed when 12.5% Ge atoms are made into vacancies. This is because removing the Ge atoms decreases the number of electrons, thus pushing the *E_F_*downward, away from the unfavorable bonding region. Although some antibonding interactions remain in Ge_1_Sb_2_Te_4_ right below the *E_F_*, they do not affect the stability of the compound significantly. In fact, this behavior is constantly observed in phase change materials, such as other GST compounds and their binary parent compounds, GeTe [56,57] and Sb_2_Te_3_ [58].

**Figure 2:**
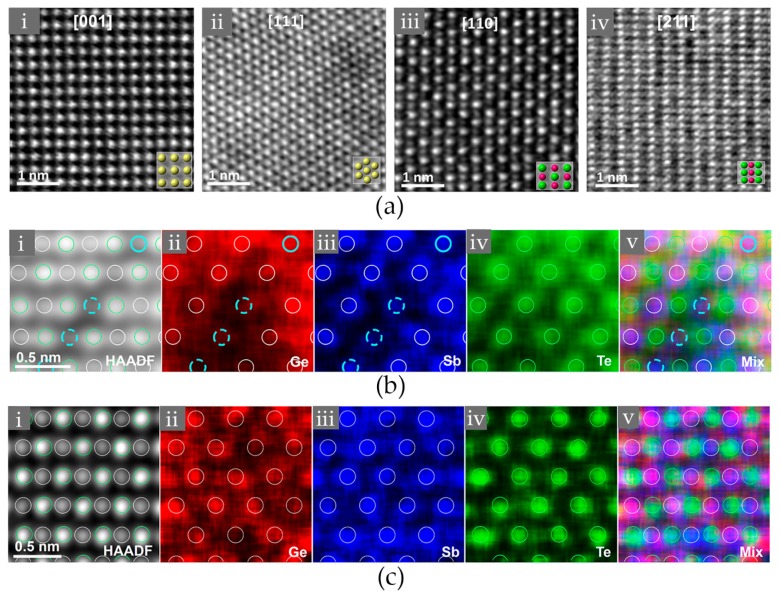
(**a**) The high angle annular dark field scanning transmission electron microscopy (HAADF-STEM) images of Ge_2_Sb_2_Te_5_ along the [001], [111], [110], and [211] crystallographic orientations. Insert in the bottom right corner of each image is the arrangement obtained from the ideal atomic model. Yellow color indicates that the atomic column includes both cation-like and anion-like atoms, while pink and green indicate the cation-like and anion-like sublattices, respectively. (**b**,**c**) The EDX chemical mappings of rocksalt Ge_2_Sb_2_Te_5_ along the [110] direction with Ge, Sb and Te being marked in red, blue and green, respectively. The mixture of these mappings is also shown. (**b**,**c**) Are taken from the different parts of the same sample. Adapted with permission from Reference [42]. © 2016 American Institute of Physics.

It is known that the high amount of vacancies, Ge and Sb atoms are randomly distributed on the cation-like sublattice. N. Yamada and co-workers firstly proposed such disordered rocksalt structure based on X-ray diffraction (XRD) data [38,43,44]. This structure was later corroborated by transmission electron microscopy experiments [42,59]. In Ref. [42], B. Zhang et al. studied the structural properties of rocksalt Ge_2_Sb_2_Te_5_ thin films comprehensively using the spherical aberration corrected (Cs-corrected) high angle annular dark field scanning transmission electron microscopy (HAADF-STEM) and the energy-dispersive X-ray (EDX) mapping experiments, as shown in Figure 2. Such method has shown to have promising capability in elucidating the crystal structures and defects of crystalline chalcogenides [60,61,62]. Several crystallographic orientations were measured, and the obtained lattice parameter ~6.0 Å is in good agreement with previous XRD data [43]. The spots in Figure 2a correspond to the contribution of the whole atomic columns along the viewing direction, and the brightness of each spot is roughly proportional to Z^2^ [63], where Z represents the average atomic number of the atomic column (here, Ge 32, Sb 51, Te 52, vacancy 0). Imaging along the [110] or [211] directions can well distinguish the anion-like sublattice from the cation-like one, where the former one shows uniform and bright spots, suggesting the full occupation of the heavier element Te, while the latter one displays varying brightness, suggesting high randomness in the cation-like sublattice. Direct observations of elemental distributions at the atomic level unambiguously clarify the presence and random occupation of atomic vacancies, as shown in Figure 2b,c. The two sets of EDX mappings were made on different parts of the same sample, and in Figure 2b, the three dashed cyan circles clearly mark out the presence of vacancies, as both Ge and Sb signals are weak. These three vacancy enriched atomic columns indicate some trend for vacancy ordering as the sample was further annealed after recrystallization, while the vacancies in other parts of the same sample still remain randomly distributed. Overall, this elemental-resolved atomic imaging experiment confirmed the disordered rocksalt structure proposed by N. Yamada and coworkers.

The presence of vacancies and compositional disorder in rocksalt GST crystals also induces distortions around the ideal lattice sites [26,64]; indeed, DFT simulations show that the average distortion is ~0.18 Å in rocksalt Ge_1_Sb_2_Te_4_ at zero temperature [45,65].

**Figure 3:**
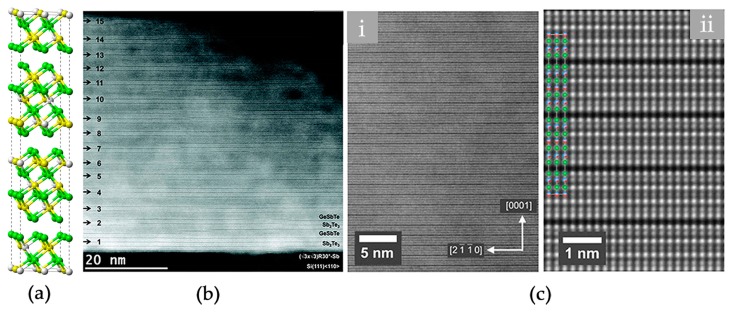
(**a**) The atomic model of hexagonal Ge_1_Sb_2_Te_4_. Chemical disorder is included for the cationic-like layers. Ge, Sb and Te atoms are rendered with grey, yellow and green spheres, respectively; (**b**) The HAADF-STEM image of hexagonal Ge_1_Sb_2_Te_4_, which was obtained by annealing a molecular beam epitaxy (MBE) grown sample at 400 °C for 30 min; (**c**) The HAADF-STEM images of hexagonal Ge_1_Sb_2_Te_4_, which was produced by metal organic vapor phase epitaxy (MOVPE) method with a growth temperature above 400 °C. The brighter dots indicate Te atomic columns, whereas darker dots are Ge/Sb atomic columns. Adapted with permission from References [66,67] © 2015 The Royal Society of Chemistry; 2016 Elsevier Ltd.

The stable crystalline phase of GST is a hexagonal structure with atomic blocks of various size held together by van der Waals (vdW) forces [68,69,70,71]. Atoms take three special positions in the *xy* plane, i.e., A = (0, 0), B = (1/3, 2/3), and C = (2/3, 1/3). The atomic blocks of Ge_1_Sb_2_Te_4_ and Ge_2_Sb_2_Te_5_ are made of 7 and 9 alternating Ge/Sb and Te layers, respectively. The blocks are terminated with Te layers, and the adjacent two Te layers are weakly coupled by vdW forces. The atomic structure of hexagonal Ge_1_Sb_2_Te_4_ is shown in Figure 3a. The periodicity of the structure is 21 atomic layers along the *z* direction. Regarding the *xy* plane, it is evidenced by both X-ray diffraction and transmission electron microscopy experiments that chemical disorder could be present in the cation-like layers [66,67], see STEM-HAADF images in Figure 3b,c. If there is no chemical disorder, two cationic-like layers should have the same brightness as the Te layers, as the atomic number of Sb (51) is very close to that of Te (52). Other possible forms of disorder in hexagonal GST include stacking faults, twinning and vacancy layer intersections, antisites and so on [72,73,74].

### 2.2. Disorder-Induced Electron Localization

**Figure 4:**
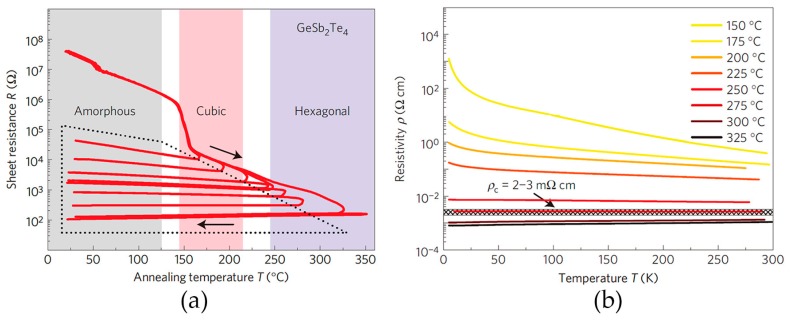
(**a**) Temperature dependence of the sheet resistance measured in a 100-nm-thick Ge_1_Sb_2_Te_4_ film. The samples were heated up quickly and annealed at different temperatures for 30 min, then were cooled down to room temperature; (**b**) Low-temperature transport measurement of Ge_1_Sb_2_Te_4_ thin films. The lowest temperature reached 5 K. Different colors of curves in (**b**) represent eight samples annealed in the range between 150 °C and 325 °C. Adapted with permission from Reference [12] © 2011 Nature Publishing Group.

What are the effects of the high amount of disorder present in GST crystals, in particular to the electrical properties? This question was thoroughly addressed by electrical transport experiments in Ref. [12], in which T. Siegrist et al. measured the sheet resistance of GST thin films annealed at different temperatures. The as-deposited GST thin film was amorphous, and the resistance at room temperature was measured to be very high, while upon heating at 150 °C, the resistance dropped drastically, as the film crystallized into the rocksalt phase, see Figure 4a. The pronounced electrical resistance contrast between the amorphous and rocksalt state can be utilized to encode digital data as “0” and “1”, and this is the mechanism that has been exploited in conventional phase change memory devices. However, it was found that upon further thermal annealing at even higher temperatures, the room temperature resistance would further reduce but in a continuous manner. For the GST annealed at 325 °C, the sheet resistance was roughly three orders of magnitude smaller than that annealed 150 °C, and could be clearly identified as a third logic state “2”. The multi-level resistance states achieved within one memory cell could enable multi-level data storage, which could increase the storage density dramatically.

The temperature coefficient of resistivity (TCR) is an important indicator of the transport properties, with negative slope (d*ρ*/d*T* < 0) representing insulating behavior while positive slope representing (d*ρ*/d*T* > 0) metallic behavior. To further clarify the transport properties, the low temperature resistivity was measured in Ref. [12]. Close to zero temperature, the resistivity of insulating solids diverges, while that of metallic solids reaches a finite value. The transport data presented in Figure 4 clearly distinguish the insulating rocksalt GST (annealed at 150 °C) from the metallic hexagonal GST (annealed at 300 °C). Both rocksalt and hexagonal GST are found to be p-type semiconducting, which stems from the presence of non-stoichiometric excess vacancies on the cation-like sublattice. The concentration of these excess vacancies corresponds to approximately 1 Ge or Sb vacancy out of 1000 atoms [75,76,77], which accounts for the high carrier concentration ~10^20^ cm^−3^ measured in the crystalline GST thin films [12] (It is noted that these excess vacancies at concentration ~0.1% are different from the intrinsic atomic vacancies that we discussed before.) In fact, no big change of the concentration of excess vacancies, and thereby the carrier concentration, was found upon thermal annealing. Thus another mechanism has to account for the huge resistance change. Instead, the carrier mobility measured by Hall and van der Pauw experiments showed an increase over 100 times upon annealing, and is therefore responsible for the pronounced contrast in electrical resistance and the MIT. The van der Pauw and Hall measurements also determined that the electron mean free path of the insulating samples was of the order of 10 Å. [12]. Since this value is much smaller than the grain size of the crystalline samples, ~100–200 Å [44], the scattering of electrons should be attributed to an intra-grain mechanism. In GST, the electron-correlation effects are weak due to the high static dielectric constant of 98, which stems from the alignment of directional p orbitals, forming resonant bonding [40,41,78]. Therefore, Mott physics is expected not to be the dominant mechanism responsible for the MIT. Instead, the large amount of atomic disorder should play a major role here. Combined with the fact that the low temperature transport data of insulating samples could be well fitted with a variable-range hopping model, it is plausible to conclude that rocksalt GST is an Anderson insulator [12,13]. Other vacancy-rich GeSbTe samples, including Ge_2_Sb_2_Te_5_, Ge_3_Sb_2_Te_6_, and Ge_1_Sb_4_Te_7_ hold the same transport features [12].

**Figure 5:**
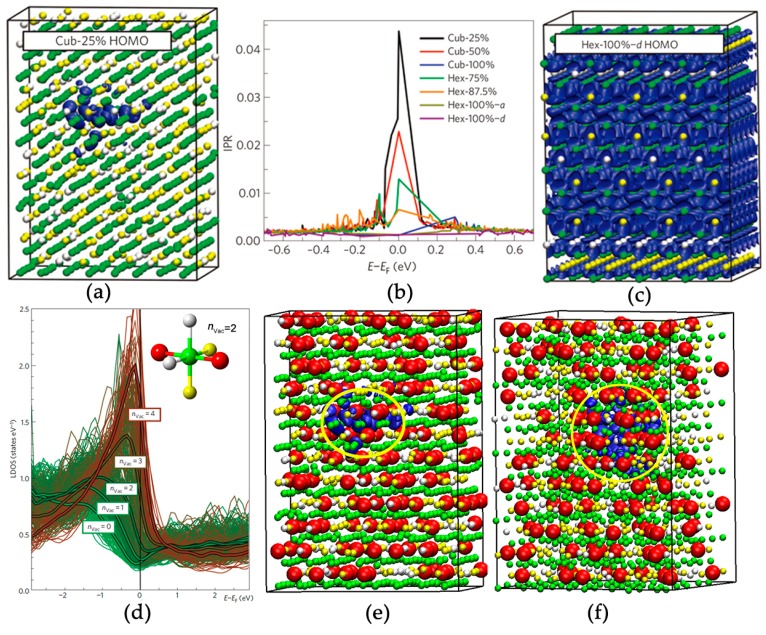
(**a**,**c**) Are the charge density distributions of the electron wave functions (blue spheres) of the disordered rocksalt Ge_1_Sb_2_Te_4_ (Cub-25%) and the perfect hexagonal Ge_1_Sb_2_Te_4_ (Hex-100%-*d*) at the Fermi level, i.e., the highest occupied molecular orbital (HOMO) states. Ge, Sb and Te atoms are rendered with grey, yellow and green spheres, respectively; (**b**) Inverse participation ratio (IPR) curves for several rocksalt and hexagonal Ge_1_Sb_2_Te_4_ models with different degree of disorder; (**d**) The local density of p states (LDOS) of the Te atoms, which are categorized according to the number of the nearest-neighbor vacant sites *n*_Vac_ (inset) with different colors. The average LDOS of each *n*_Vac_ group is shown as a thick line in the corresponding color. The atomic vacancies in (a) are highlighted with big red balls and are shown in (**e**). The yellow circle marks a vacancy cluster. (**f**) The highest occupied molecular orbital (HOMO) state of a rocksalt Ge_1_Sb_2_Te_4_ model with a manually created vacancy cluster, which clearly localizes the electron wave functions. Adapted with permission from Reference [15] © 2012 Nature Publishing Group.

As discussed above, the intra-grain atomic disorder in GST includes (a) the very high concentration of atomic vacancies; (b) the statistical distribution of Ge, Sb and atomic vacancies on the cation-like sublattice; (c) non-negligible atomic displacements away from the ideal rocksalt lattice sites; and (d) some small amount of other defects, such as excess vacancies, anti-site defects, etc. From experiments, it is very difficult to disentangle the contributions of these defects, and capture the form of disorder that is mainly responsible for Anderson localization. In Ref. [15], W. Zhang et al. performed large-scale DFT simulations to access the electronic structure and the role of disorder in crystalline GST. In Figure 5a, an orthorhombic supercell of rocksalt GST is shown, in which the atomic vacancies, Ge and Sb atoms were placed randomly. The z direction of the supercell is parallel to the [111] crystallographic orientation of rocksalt GST, and the cation-like and anion-like sublattices are well separated along the z direction. The model contained 1008 atoms, and the shortest cell parameter was over 25 Å, which, as we shall see, is significantly larger than the typical localization length. Upon geometry optimization, atoms deviated from the ideal lattice sites with an average displacement value close to 0.2 Å, in line with previous findings [45,65].

The Kohn–Sham eigenstates close to the Fermi level were analyzed, and a key parameter that characterizes the degree of localization, the Inverse Participation Ratio (IPR), was computed for these electronic states. Finite IPRs indicate that the electronic states are localized, while the IPRs of extended states are close to zero for sufficiently large system sizes. For models with ~1000 atoms, the IPRs of extended states are of the order of 10^−3^, and such value should fully vanish when the system becomes infinitely large. In Figure 5b, the IPRs of the electronic states of disordered rocksalt GST at the Fermi level are an order of magnitude larger than 10^−3^, revealing them to be localized states. These IPRs correspond to a group of ~25–60 atoms, giving a localization length ~10 Å, in line with that obtained from transport experiments. The highest occupied molecular orbital (HOMO) state of the most disordered rocksalt GST is plotted in Figure 5a. The electron wave function is found to be very well localized in the upper side of the model (blue spheres). A detailed structural analysis showed that the concentration of atomic vacancies in this region was quite high, almost twice larger than the average value. The atomic vacancies are highlighted with bigger red balls in Figure 5e. The higher vacancy concentration in this region corresponds to several Te atoms with 2–4 vacant neighbors, and was denoted as “vacancy cluster” in Ref. [15]. It needs to be stressed that “vacancy clusters” are not voids, as many atoms are present inside this region. To verify this claim, a rocksalt GST model with a manually arranged “vacancy cluster” was generated and computed, and the electron wave function of the HOMO state appeared in this vacancy-rich region as expected. A thorough analysis of the local density of states (LDOS) of Te atoms according to their nearest-neighbor configuration further confirmed the essential role of atomic vacancies in localizing electrons. As shown in Figure 5d, a pronounced increase of LDOS as a function of the nearest-neighbor vacant sites *n*_Vac_ of Te atoms can be observed. It is noted that this analysis was carried out on unrelaxed structures of rocksalt GST (up to 4096 lattice sites, 16 × 16 × 16 supercell ~5 × 5 × 5 nm^3^). Relaxed models show similar trends. Therefore, we can conclude that atomic displacements are not important to the electron localization in GST.

As to be discussed below, compositional disorder alone cannot induce any electron localization either. Excess vacancies were shown to shift the Fermi level towards lower energy of the valence band, but not away from the localization region. A precise determination of the mobility edge separating localized from extended states is challenging, as the localization length diverges at the transition point. Recently, small fractions of antisite defects, yielding some Te–Te and Ge(Sb)–Ge(Sb) homopolar bonds, were found in recrystallized Ge_2_Sb_2_Te_5_ obtained by DFT-based molecular dynamics (DFMD) simulations [79,80,81,82,83,84]. A thorough understanding of the effects of antisite defects on the localization properties is anticipated, nevertheless, in a recent study on pressuring rocksalt Ge_1_Sb_2_Te_4_ at elevated temperatures, pairwise antisites were found but were shown to be ineffective for the localization properties of the occupied electronic states at the Fermi level [85]. To conclude, the presence and random distribution of a high amount of atomic vacancies in crystalline GST would unavoidably result in the formation of “vacancy clusters”, giving rise to the localization of electrons in crystalline GST. Spin–orbit coupling (SOC) effects are shown to be significant in crystalline GST [86,87,88,89,90,91], while they do not alter the localization properties discussed above [16].

### 2.3. Disorder-Driven Metal–Insulator Transition

**Figure 6:**
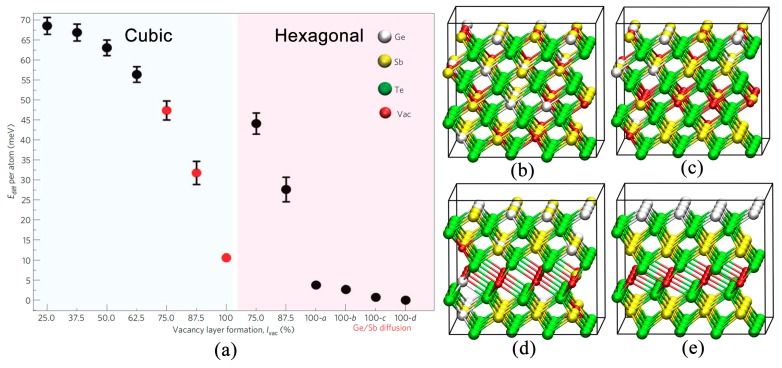
(**a**) The energy trend of Ge_1_Sb_2_Te_4_ with the reduction of disorder and the formation of vacancy layers. The atomic vacancies migrate from cation-like layers to some specific cation-like layers (3 out of 24 atomic layers in a rocksalt Ge_1_Sb_2_Te_4_ supercell model). The vacancy concentration on these layers increases from 25 to 100%. The red point at 100% in the cubic region indicates the ordered cubic phase with fully formed “vacancy layers”. Chemical Ge/Sb disorder on the cation-like layers is included; (**b**–**e**) The atomic structure of cubic and hexagonal GST models: (**b**) Cub-25% (**c**) Cub-50% (**d**) Hex-75% (**e**) Hex-100%-*d.* The four hexagonal models differ in the degree of chemical Ge/Sb disorder on the cation-like layers. Small red spheres indicate vacancies. Only parts of full models are shown, for the sake of clarity. Adapted with permission from Reference [15] © 2012 Nature Publishing Group.

After elucidating the essential role of vacancies in inducing electron localization, it is important to investigate the evolution of vacancies and its effects on the electronic structure of GST. The transport experiments in Ref. [12] showed a clear sign change of the TCR slope and a finite resistivity value near zero K for the GST samples annealed at 300 °C and above, suggesting a MIT. Comprehensive materials modeling and DFT simulations have shed light onto the underlying mechanism of this MIT [15]. For the initial rocksalt GST, all the cation-like layers contained 25% Ge, 50% Sb and 25% vacancies. To enable a gradual structural change to hexagonal GST with van der Waals gaps (we refer to these gaps as “vacancy layers” starting from now on), 3 out of 12 cation-like layers in rocksalt GST were selected, and were made vacancy-rich gradually, starting from 25% until 100% (this percentage is denoted with *l*_Vac_). The total number of atoms for all the models is the same, thereby the increase in atomic vacancies in the selected cation-like layers corresponds to a decrease in vacancies in the other cation-like layers. The corresponding total energy of the relaxed models is plotted as a function of *l*_Vac_, see Figure 6a. A gradual reduction of the total energy suggests this vacancy ordering process to be a reasonable path for the structural evolution upon thermal annealing. When *l*_Vac_ reaches a certain value, between 60% and 70%, the hexagonal stacking sequence becomes energetically more favorable, suggesting that a cubic–hexagonal structural (cub–hex) transition would occur. Upon further vacancy ordering *l*_Vac_ > 87%, a MIT is triggered. As shown in Figure 5b, the IPRs of hexagonal GST with *l*_Vac_ = 100% decreased down to ~10^−3^, indicating extended states. The HOMO state of hexagonal GST without any disorder is shown in Figure 5c, and the electron wave function spreads over the whole space. It is noted that chemical Ge/Sb disorder alone on the cation-like layers could not alter the delocalized nature of electrons, once the atomic vacancies are fully ordered into layers. Several snapshots of the GST models with different *l*_Vac_ are shown in Figure 6c–e. For the sake of clarity, only part of the models and one “vacancy layer” are displayed. In summary, the ordering of atomic vacancies firstly triggers a structural transition and then a MIT. The two processes can be well separated.

**Figure 7:**
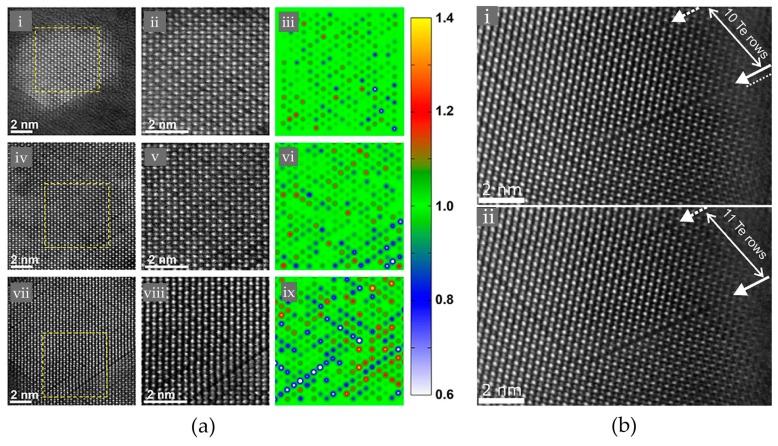
(**a**) The HAADF-STEM images of rocksalt Ge_2_Sb_2_Te_5_ annealed at 150 °C for 2 min (i–iii), 160 °C for 30 min (iv–vi), and 180 °C for 30 min (vii–ix). The zoom-in views and the corresponding normalization maps (only the cation-like atomic columns were considered) of the boxed regions in (i), (iv) and (vii) are shown in (ii–iii), (v–vi) and (viii–ix), respectively; (**b**) The MAADF-STEM images of rocksalt Ge_2_Sb_2_Te_5_ under focused electron beam irradiation. The movement of a vacancy-rich cation-like layer was observed and was marked by the arrows in (i) and (ii). Adapted with permission from References [42,92]. © 2016 American Institute of Physics; 2016 Elsevier Ltd.

The predicted vacancy ordering process was demonstrated by ex-situ STEM experiments. In Ref. [42], STEM experiments were performed on sputtered Ge_2_Sb_2_Te_5_ samples annealed at 150 °C, 160 °C and 180 °C, and the corresponding HAADF images were presented in Figure 7a. Initially, atomic vacancies distributed randomly, while upon thermal annealing, some long and dark patches formed along the [111] crystallographic orientation of the rocksalt phase. A color-scheme normalized map of the HAADF images with only the cation-like sites was developed to give a better view of this process. For the sample annealed at 150 °C for 2 min, the concentration of atomic vacancies fluctuated from column to column, and some vacancy-rich columns could be found. For the samples annealed at 160 °C and 180 °C for 30 min, more and more vacancy-rich columns were formed and connected. At this early stage of the vacancy ordering process, all the samples remained insulating. The exposure to electron beam is unavoidable in STEM experiments; nevertheless, it was shown that electron beam irradiation within limited data collection time could not induce any structural change in Reference [37]. A. Lotnyk et al. also reported [92] that the movement of a vacancy-rich layer was detected under focused electron beam, as shown in Figure 7b.

### 2.4. Metal–Insulator Transition without Cub–Hex Structural Transition

Although it is already clear from the above transport experiments and DFT simulations that the cub–hex structural transition does not play a crucial role in inducing the MIT observed in crystalline GST, we can still wonder if it is possible to further disentangle these two transitions. The answer is yes. From some early DFT simulations, an ordered cubic structure with complete “vacancy layers” was proposed by S.-M. Sun et al. [70] and J. Da Silva et al. [71], as the ordered cubic phase was found to be energetically more favorable than the disordered rocksalt phase through DFT simulations. From Figure 6a, one can see that the ordered cubic GST (with Ge/Sb disorder) is about 55 meV/atom lower in energy than the disordered rocksalt GST. In fact, such model is energetically comparable to hexagonal GST (with Ge/Sb disorder), as the energy difference is only about 5 meV/atom. Such small energy difference suggests that this ordered cubic phase might be produced experimentally. Very recently, several groups reported the existence of ordered cubic GST in experiments, which were obtained by molecular beam epitaxy (MBE) [93], pulsed laser deposition [94,95], or by the application of a designed electric current [96].

**Figure 8:**
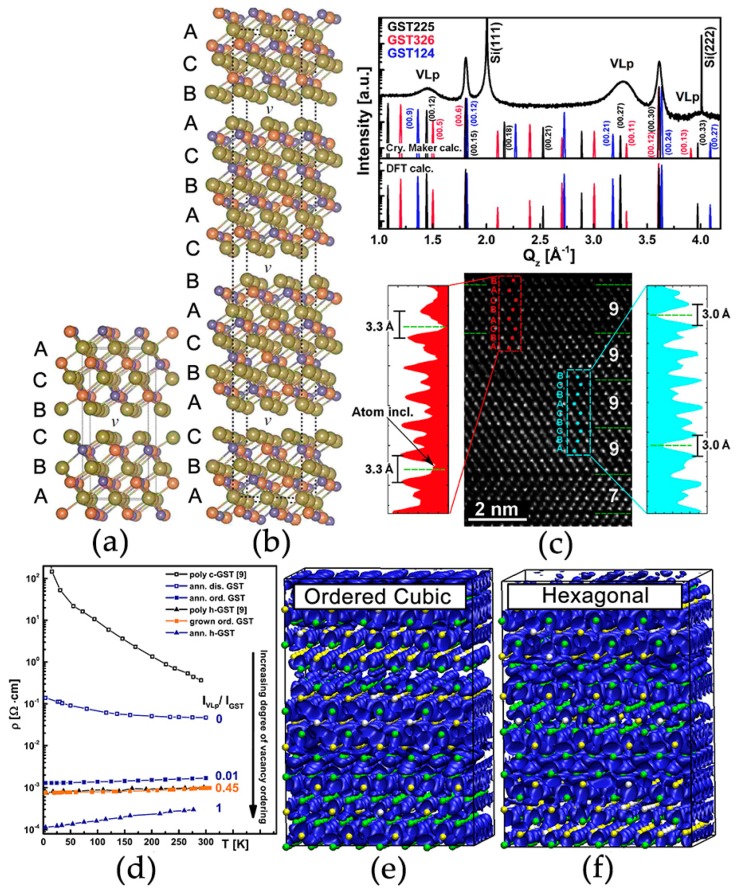
(**a**,**b**) The atomic structure of hexagonal and ordered cubic Ge_2_Sb_2_Te_5_. The stacking sequence of Te layers is labelled with A, B and C; (**c**) The XRD curve and HAADF-STEM image of ordered cubic Ge_2_Sb_2_Te_5_ grown by MBE method. “Vacancy layers” (VLs) appear every 7–9 atomic layers, marked by dashed green lines. The cubic and hexagonal stacking coexists in this MBE grown sample, boxed by red and cyan dashed lines; (**d**) Resistivity as a function of temperature for epitaxial and sputtered GST samples. Empty and filled squares denote the disordered rocksalt phase and ordered cubic phase, while triangles denote the hexagonal phase; (**e**,**f**) Are snapshots of the HOMO state of the ordered cubic and hexagonal GST. Chemical disorder Ge/Sb on the cation-like layers is included for both models. Adapted with permission from References [12,15,71,93] © 2008 American Physical Society; 2011, 2012, 2016 Nature Publishing Group.

The major difference between ordered cubic and hexagonal phase is the stacking sequence. We take Ge_2_Sb_2_Te_5_ as example. The sequence of Te layers in hexagonal Ge_2_Sb_2_Te_5_ is –ABCBC–, while that of ordered cubic phase is –ABCABCABCABC–, as marked in Figure 8a,b. V. Bragaglia et al. [93] produced cubic Ge_2_Sb_2_Te_5_ using MBE, but some unconventional peaks in XRD patterns were consistently detected. In comparison with XRD pattern simulations of ordered cubic models (Figure 8c), these peaks were identified to be vacancy layer peaks (VLp). STEM experiments provided more direct evidence on the ordered cubic phase. The red and cyan dashed lines in the HAADF image (see Figure 8c) marked out the cubic and hexagonal stacking of GST, proving the co-existence of the two phases in the MBE-grown samples. The low-temperature transport data showed that the MBE samples were metallic (Figure 8d), the same as the hexagonal phase. DFT simulations on ordered cubic GST also revealed that the electronic states near the Fermi level were completely delocalized (Figure 5b and Figure 8e), giving rise to the observed metallic behavior [15]. This extended nature is the same as that in the hexagonal phase (Figure 8f). It is noted that in both models, the Ge/Sb on cation-like layers were considered, and were shown to be ineffective to the electronic structure. Now, we can safely conclude that the MIT in crystalline GST is purely electronically driven, and the crystallographic structural transition is irrelevant for the MIT.

## 3. Conclusions

To conclude, we have provided a comprehensive overview of Anderson localization of electrons and disorder-driven metal–insulator transitions in a technologically-important material, the Ge_1_Sb_2_Te_4_ phase change material. The existence of strong atomic disorder has been unequivocally demonstrated by element-resolved atomic structure imaging experiments [42], DFT simulations and quantum chemistry bonding analysis [45]. Electron localization due to strong disorder has been clearly identified by low-temperature transport experiments [12,13], and its origin has been revealed by DFT simulations. These simulations showed that the fluctuations in the distribution of atomic vacancies and the consequent formation of “vacancy clusters” lead to the localization of electrons near the Fermi level [15]. Upon thermal annealing, “vacancy clusters” dissolve and atomic vacancies diffuse into specific cation-like layers, forming “vacancy layers”. This vacancy ordering process firstly leads to a structural transition from the metastable cubic rocksalt phase to the stable hexagonal phase, and then triggers a metal–insulator transition, prior to the complete formation of “vacancy layers”. For such models, the electronic states near the Fermi level become fully extended [12,15]. Cubic GST samples with ordered “vacancy layers” have been produced experimentally very recently [93,94,95] and measured to be metallic [93,96], which confirms the DFT predictions in Ref. [15]. The metal–insulator transition between disordered and ordered GST proves that this transition is purely electronically-driven, and is distinct from ordinary crystallographic transitions. It is worth mentioning that another type of disorder-induced MIT was reported in phase change nanowires, where non-local disorder, such as dislocations and antiphase boundaries, were found to play a dominant role [97,98]. The accumulation of these defects was driven by electric wind force, which eventually led to amorphization. Metal–insulator transitions were found along this non-thermal phase transition path [97,98].

To enable multi-level data storage in phase change memory, the conventional route is to engineer the amorphous–crystalline volume ratio of memory cells [19,20] to achieve distinguishable multi-level resistance states. However, such route suffers from the resistance drift issue of the amorphous part, which stems from the intrinsic aging property of the materials [99,100,101,102,103,104]. In addition, when the size of phase change memory devices is scaled down to few nanometers [105,106], such volume ratio strategy is no longer feasible to support many intermediate resistance states. Disorder control, in particular in crystalline GST, provides an alternative way to enable multi-level data storage [12,15]. The current studies show a successive vacancy ordering process within crystalline GeSbTe compounds upon thermal annealing [42], while since the annealing temperature is low ~150–300 °C, the annealing time is thus very long ~few min. Much faster switching speed is of necessity for practical applications. Up to date, it is unclear how fast the vacancy ordering process can occur at very high temperature ~400–500 °C, corresponding to very high electrical pulses in devices. Nevertheless, DFT simulations already give some positive hints. It was predicted that the barrier of interlayer vacancy diffusion in GST crystals is not very high ~0.8–1.0 eV, and several such vacancy diffusion events were found in very short time scale ~50 picoseconds at 500 °C [17,42]. This transition barrier can be further reduced by pressure [85]. It is highly desirable to thoroughly test the vacancy ordering speed within the crystalline states in nanoscale phase change devices.

Owing to the importance of disorder control in GST from both the application and fundamental research perspectives, this subfield is under very active investigation. In addition to thermal annealing, pressure [85,107,108,109,110,111], strain [112,113], chemical composition [14,16], ion bombardment [114], focused electron beam irradiation [92], electric field [115,116], voltage pulses [117,118,119], laser and photonic excitation [120,121], and interface template [93,122,123] are shown to be effective approaches in tuning disorder in GST and related compounds. It is also worth mentioning that the remaining disorder in ultrathin hexagonal GST films ~7–14 nm would result in a weak anti-localization phenomenon. The disorder includes stacking faults, bi-layer defects and chemical disorder, etc., [66,124] however, the role of these defects in affecting the transport properties is still unclear, which deserves further investigations [125].

Before closing, we note that, very recently, clear evidence for Anderson localization of electrons due to strong disorder has also been observed in other chalcogenide compounds, such as ultrathin (Bi_1−*x*_Sb*_x_*)_2_Te_3_ films [126] and Li*_x_*Fe_7_Se_8_ bulk single crystals [127]. We can foresee more and more crystalline compounds with strong disorder and weak electron correlation to be identified, which shall further elucidate the role of disorder in tailoring the electrical transport properties of crystalline solids. We believe that these crystalline solids with tunable electrical properties controlled by atomic disorder will lead to the design of novel electronic devices for various applications.

## References

[1] Ashcroft N.W., Mermin N.D. (1976). Solid State Physics.

[2] De Boer J.H., Verwey E.J.W. (1937). Semi-conductors with partially and with completely filled 3d-lattice bands. Proc. Phys. Soc..

[3] Mott N.F., Peierls R. (1937). Discussion of the paper by de Boer and Verwey. Proc. Phys. Soc..

[4] Mott N.F. (1968). Metal-Insulator Transition. Rev. Mod. Phys..

[5] Mott N.F. (1982). Review lecture: Metal-Insulator Transitions. Proc. R. Soc. Lond. A.

[6] Anderson P.W. (1958). Absence of diffusion in certain random lattices. Phys. Rev..

[7] Abrahams E., Anderson P.W., Licciardello D.C., Ramakrishnan T.V. (1979). Scaling theory of localization: Absence of quantum diffusion in two dimensions. Phys. Rev. Lett..

[8] Abrahams E. (2010). 50 Years of Anderson Localization.

[9] Rosenbaum T.F., Andres K., Thomas G.A., Bhatt R.N. (1980). Sharp insulator transition in a random solid. Phys. Rev. Lett..

[10] Rosenbaum T.F., Milligan R., Paalanen M., Thomas G.A., Bhatt R.N., Lin W. (1983). Metal-insulator transition in a doped semiconductor. Phys. Rev. B.

[11] Shklovskii B.I., Efros A.L. (1984). Electronic Properties of Doped Semiconductors.

[12] Siegrist T., Jost P., Volker H., Woda M., Merkelbach P., Schlockermann C., Wuttig M. (2011). Disorder-induced localization in crystalline phase-change materials. Nat. Mater..

[13] Volker H., Jost P., Wuttig M. (2015). Low-Temperature Transport in Crystalline Ge_1_Sb_2_Te_4_. Adv. Funct. Mater..

[14] Jost P., Volker H., Poitz A., Poltorak C., Zalden P., Schaefer T., Lange F., Schmidt R.M., Hollaender B., Wirtssohn M.R. (2015). Disorder-Induced Localization in Crystalline Pseudo-Binary GeTe–Sb_2_Te_3_ Alloys between Ge_3_Sb_2_Te_6_ and GeTe. Adv. Funct. Mater..

[15] Zhang W., Thiess A., Zalden P., Zeller R., Dederichs P.H., Raty J.Y., Wuttig M., Blügel S., Mazzarello R. (2012). Role of vacancies in metal-insulator transitions of crystalline phase-change materials. Nat. Mater..

[16] Zhang W., Wuttig M., Mazzarello R. (2015). Effects of stoichiometry on the transport properties of crystalline phase-change materials. Sci. Rep..

[17] Zhang W. (2014). Ab Initio Investigation of Phase Change Materials: Structural, Electronic and Kinetic Properties. Ph.D. Thesis.

[18] Wuttig M., Yamada N. (2007). Phase-change materials for rewriteable data storage. Nat. Mater..

[19] Wong H.S.P., Raoux S., Kim S.B., Liang J., Reifenberg J.P., Rajendran B., Asheghi M., Goodson K.E. (2010). Phase Change Memory. Proc. IEEE.

[20] Raoux S., Welnic W., Ielmini D. (2010). Phase change materials and their application to nonvolatile memories. Chem. Rev..

[21] Zhu L., Zhou J., Guo Z., Sun Z. (2015). An overview of materials issues in resistive random access memory. J. Materiomics.

[22] Yamada N., Kojima R., Hisada K., Mihara T., Tsuchino A., Fujinoki N., Birukawa M., Matsunaga T., Yasuda N., Fukuyama Y. (2013). Phase-Change Nanodot Material for an Optical Memory. Adv. Opt. Mater..

[23] Ríos C., Stegmaier M., Hosseini P., Wang D., Scherer T., Wright C.D., Bhaskaran H., Pernice W.H.P. (2015). Integrated all-photonic non-volatile multi-level memory. Nat. Photonics.

[24] Wright C.D., Liu Y., Kohary K.I., Aziz M.M., Hicken R.J. (2011). Arithmetic and biologically-inspired computing using phase-change materials. Adv. Mater..

[25] Wright C.D., Wang L., Aziz M.M., Diosdado J.A.V., Ashwin P. (2012). Phase-change processors, memristors and memflectors. Phys. Status Solidi B.

[26] Shamoto S. (2005). Large displacement of germanium atoms in crystalline Ge_2_Sb_2_Te_5_. Appl. Phys. Lett..

[27] Tuma T., Pantazi A., Le Gallo M., Sebastian A., Eleftheriou E. (2016). Stochastic phase-change neurons. Nat. Nanotechnol..

[28] Li Y., Zhong Y., Xu L., Zhang J., Xu X., Sun H., Miao X. (2013). Ultrafast Synaptic Events in a Chalcogenide. Memristor.

[29] Song W.-D., Shi L.-P., Miao X.-S., Chong C.-T. (2008). Synthesis and Characteristics of a Phase-Change Magnetic Material. Adv. Mater..

[30] Zhang W., Ronneberger I., Li Y., Mazzarello R. (2012). Magnetic properties of crystalline and amorphous phase-change materials doped with 3d impurities. Adv. Mater..

[31] Li Y., Mazzarello R. (2012). Magnetic contrast in phase-change materials doped with Fe impurities. Adv. Mater..

[32] Liu J.D., Miao X.S., Tong F., Luo W., Xia Z.C. (2013). Ferromagnetism and electronic transport in epitaxial Ge_1 − *x*_Fe*_x_*Te thin film grown by pulsed laser deposition. Appl. Phys. Lett..

[33] Zhang W., Ronneberger I., Li Y., Mazzarello R. (2014). Ab initio investigation of crystalline and amorphous GeTe doped with magnetic impurities. Sci. Adv. Mater..

[34] Hosseini P., Wright C.D., Bhaskaran H. (2014). An optoelectronic framework enabled by low-dimensional phase-change films. Nature.

[35] Zhang W., Deringer V.L., Dronskowski R., Mazzarello R., Ma E., Wuttig M. (2015). Density functional theory guided advances in phase-change materials and memories. MRS Bull..

[36] Deringer V.L., Dronskowski R., Wuttig M. (2015). Microscopic Complexity in Phase-Change Materials and its Role for Applications. Adv. Funct. Mater..

[37] Yamada N., Ohno E., Nishiuchi K., Akahira N., Takao M. (1991). Rapid-phase transitions of GeTe-Sb2Te3 pseudobinary amorphous thin films for an optical disk memory. J. Appl. Phys..

[38] Yamada N., Matsunaga T. (2000). Structure of laser-crystallized Ge_2_Sb_2+x_Te_5_ sputtered thin films for use in optical memory. J. Appl. Phys..

[39] Simpson R.E., Krbal M., Fons P., Kolobov A.V., Tominaga J., Uruga T., Tanida H. (2010). Toward the ultimate limit of phase change in Ge_2_Sb_2_Te_5_. Nano Lett..

[40] Shportko K., Kremers S., Woda M., Lencer D., Robertson J., Wuttig M. (2008). Resonant bonding in crystalline phase-change materials. Nat. Mater..

[41] Lencer D., Salinga M., Grabowski B., Hickel T., Neugebauer J., Wuttig M. (2008). A map for phase-change materials. Nat. Mater..

[42] Zhang B., Zhang W., Shen Z.J., Chen Y.J., Li J.X., Zhang S.B., Zhang Z., Wuttig M., Mazzarello R., Ma E. (2016). Element-resolved atomic structure imaging of rocksalt Ge_2_Sb_2_Te_5_ phase-change material. Appl. Phys. Lett..

[43] Yamada N. (1996). Erasable Phase-Change Optical Materials. MRS Bull..

[44] Matsunaga T., Yamada N. (2004). Structural investigation of GeSb_2_Te_4_: A high-speed phase-change material. Phys. Rev. B.

[45] Wuttig M., Lusebrink D., Wamwangi D., Welnic W., Gillessen M., Dronskowski R. (2007). The role of vacancies and local distortions in the design of new phase-change materials. Nat. Mater..

[46] Kohn W., Sham L.J. (1965). Self-Consistent Equations Including Exchange and Correlation Effects. Phys. Rev..

[47] Hohenberg P., Kohn W. (1964). Inhomogeneous Electron Gas. Phys. Rev..

[48] Martin R.M. (2004). Electronic Structure: Basic Theory and Practical Methods.

[49] Dronskowski R., Blöchl P.E. (1993). Crystal orbital Hamilton populations (COHP): Energy-resolved visualization of chemical bonding in solids based on density-functional calculations. J. Phys. Chem..

[50] El-Mellouhi F., Mousseau N., Ordejon P. (2004). Sampling the diffusion paths of a neutral vacancy in silicon with quantum mechanical calculations. Phys. Rev. B.

[51] Deringer V.L., Tchougreeff A.L., Dronskowski R. (2011). Crystal orbital Hamilton population (COHP) analysis as projected from plane-wave basis sets. J. Phys. Chem. A.

[52] Maintz S., Deringer V.L., Tchougréeff A.L., Dronskowski R. (2013). Analytic projection from plane-wave and PAW wavefunctions and application to chemical-bonding analysis in solids. J. Comput. Chem..

[53] Maintz S., Deringer V.L., Tchougreeff A.L., Dronskowski R. (2016). LOBSTER: A tool to extract chemical bonding from plane-wave based DFT. J. Comput. Chem..

[54] Deringer V.L., Zhang W., Rausch P., Mazzarello R., Wuttig M., Dronskowski R. (2015). A chemical link between Ge-Sb-Te and In-Sb-Te phase-change materials. J. Mater. Chem. C.

[55] Wang Y.C., Zhang W., Wang L.Y., Zhuang Z., Ma E., Li J., Shan Z.W. (2016). In situ TEM study of deformation-induced crystalline-to-amorphous transition in silicon. NPG Asia Mater..

[56] Waghmare U.V., Spaldin N.A., Kandpal H.C., Seshadri R. (2003). First-principles indicators of metallicity and cation off-centricity in the IV-VI rocksalt chalcogenides of divalent Ge, Sn, and Pb. Phys. Rev. B.

[57] Deringer V.L., Zhang W., Lumeij M., Maintz S., Wuttig M., Mazzarello R., Dronskowski R. (2014). Bonding nature of local structural motifs in amorphous GeTe. Angew. Chem. Int. Ed..

[58] Stoffel R.P., Deringer V.L., Simon R.E., Hermann R.P., Dronskowski R. (2015). A density-functional study on the electronic and vibrational properties of layered antimony telluride. J. Phys. Condens. Matter.

[59] Ross U., Lotnyk A., Thelander E., Rauschenbach B. (2014). Direct imaging of crystal structure and defects in metastable Ge_2_Sb_2_Te_5_ by quantitative aberration-corrected scanning transmission electron microscopy. Appl. Phys. Lett..

[60] Jiang Y., Wang Y., Sagendorf J., West D., Kou X., Wei X., He L., Wang K.L., Zhang S., Zhang Z. (2013). Direct atom-by-atom chemical identification of nanostructures and defects of topological insulators. Nano Lett..

[61] Rao F., Song Z., Cheng Y., Liu X., Xia M., Li W., Ding K., Feng X., Zhu M., Feng S. (2015). Direct observation of titanium-centered octahedra in titanium-antimony-tellurium phase-change material. Nat. Commun..

[62] Zheng Y., Xia M., Cheng Y., Rao F., Ding K., Liu W., Jia Y., Song Z., Feng S. (2016). Direct observation of metastable face-centered cubic Sb_2_Te_3_ crystal. Nano Res..

[63] Pennycook S.J., Nellist P.D. (2011). Scanning Transmission Electron Microscopy Imaging and Analysis.

[64] Kolobov A.V., Fons P., Frenkel A.I., Ankudinov A.L., Tominaga J., Uruga T. (2004). Understanding the phase-change mechanism of rewritable optical media. Nat. Mater..

[65] Wełnic W., Pamungkas A., Detemple R., Steimer C., Blügel S., Wuttig M. (2005). Unravelling the interplay of local structure and physical properties in phase-change materials. Nat. Mater..

[66] Momand J., Wang R., Boschker J.E., Verheijen M.A., Calarco R., Kooi B.J. (2015). Interface formation of two- and three-dimensionally bonded materials in the case of GeTe-Sb_2_Te_3_ superlattices. Nanoscale.

[67] Hardtdegen H., Rieß S., Schuck M., Keller K., Jost P., Du H., Bornhöfft M., Schwedt A., Mussler G., Ahe M.v.d. (2016). A model structure for interfacial phase change memories: Epitaxial trigonal Ge_1_Sb_2_Te_4_. J. Alloys Compd..

[68] Kooi B.J., De Hosson J.T.M. (2002). Electron diffraction and high-resolution transmission electron microscopy of the high temperature crystal structures of Ge*_x_*Sb_2_Te_3 + *x*_ (*x* = 1,2,3) phase change material. J. Appl. Phys..

[69] Kooi B.J., Groot W.M.G., De Hosson J.T.M. (2004). In situ transmission electron microscopy study of the crystallization of Ge_2_Sb_2_Te_5_. J. Appl. Phys..

[70] Sun Z.M., Zhou J., Ahuja R. (2007). Unique Melting Behavior in Phase-Change Materials for Rewritable Data Storage. Phys. Rev. Lett..

[71] Da Silva J., Walsh A., Lee H. (2008). Insights into the structure of the stable and metastable (GeTe)m(Sb_2_Te_3_)n compounds. Phys. Rev. B.

[72] Momand J., Lange F.R.L., Wang R., Boschker J.E., Verheijen M.A., Calarco R., Wuttig M., Kooi B.J. (2016). Atomic stacking and van-der-Waals bonding in GeTe–Sb_2_Te_3_ superlattices. J. Mater. Res..

[73] Thelander E., Gerlach J.W., Ross U., Lotnyk A., Rauschenbach B. (2014). Low temperature epitaxy of Ge-Sb-Te films on BaF_2_ (111) by pulsed laser deposition. Appl. Phys. Lett..

[74] Ross U., Lotnyk A., Thelander E., Rauschenbach B. (2016). Microstructure evolution in pulsed laser deposited epitaxial Ge-Sb-Te chalcogenide thin films. J. Alloys Compd..

[75] Caravati S., Bernasconi M., Kühne T.D., Krack M., Parrinello M. (2009). First principles study of crystalline and amorphous Ge_2_Sb_2_Te_5_ and the effects of stoichiometric defects. J. Phys. Condens. Matter.

[76] Sun Z., Pan Y., Zhou J., Sa B., Ahuja R. (2011). Origin of p-type conductivity in layered nGeTe·mSb_2_Te_3_ chalcogenide semiconductors. Phys. Rev. B.

[77] Edwards A., Pineda A., Schultz P., Martin M., Thompson A., Hjalmarson H., Umrigar C. (2006). Electronic structure of intrinsic defects in crystalline germanium telluride. Phys. Rev. B.

[78] Chen C., Jost P., Volker H., Kaminski M., Wirtssohn M., Engelmann U., Krüger K., Schlich F., Schlockermann C., Lobo R.P.S.M. (2017). Dielectric properties of amorphous phase-change materials. Phys. Rev. B.

[79] Lee T.H., Elliott S.R. (2011). Ab Initio Computer Simulation of the Early Stages of Crystallization: Application to Ge_2_Sb_2_Te_5_ Phase-Change Materials. Phys. Rev. Lett..

[80] Skelton J.M., Loke D., Lee T.H., Elliott S.R. (2013). Structural insights into the formation and evolution of amorphous phase-change materials. Phys. Status Solidi B.

[81] Kalikka J., Akola J., Larrucea J., Jones R.O. (2012). Nucleus-driven crystallization of amorphous Ge_2_Sb_2_Te_5_: A density functional study. Phys. Rev. B.

[82] Kalikka J., Akola J., Jones R.O. (2014). Simulation of crystallization in Ge_2_Sb_2_Te_5_: A memory effect in the canonical phase-change material. Phys. Rev. B.

[83] Kalikka J., Akola J., Jones R.O. (2016). Crystallization processes in the phase change material Ge_2_Sb_2_Te_5_: Unbiased density functional/molecular dynamics simulations. Phys. Rev. B.

[84] Ronneberger I., Zhang W., Eshet H., Mazzarello R. (2015). Crystallization properties of the Ge_2_Sb_2_Te_5_ phase-change compound from advanced simulations. Adv. Funct. Mater..

[85] Xu M., Zhang W., Mazzarello R., Wuttig M. (2015). Disorder Control in Crystalline GeSb_2_Te_4_ using High Pressure. Adv. Sci..

[86] Pauly C., Liebmann M., Giussani A., Kellner J., Just S., Sánchez-Barriga J., Rienks E., Rader O., Calarco R., Bihlmayer G. (2013). Evidence for topological band inversion of the phase change material Ge_2_Sb_2_Te_5_. Appl. Phys. Lett..

[87] Sa B., Zhou J., Sun Z., Tominaga J., Ahuja R. (2012). Topological insulating in GeTe/Sb_2_Te_3_ phase-change superlattice. Phys. Rev. Lett..

[88] Sa B., Zhou J., Song Z., Sun Z., Ahuja R. (2011). Pressure-induced topological insulating behavior in the ternary chalcogenide Ge2Sb2Te5. Phys. Rev. B.

[89] Kim J., Kim J., Jhi S.-H. (2010). Prediction of topological insulating behavior in crystalline Ge-Sb-Te. Phys. Rev. B.

[90] Kim J., Kim J., Kim K.S., Jhi S.H. (2012). Topological Phase Transition in the Interaction of Surface Dirac Fermions in Heterostructures. Phys. Rev. Lett..

[91] Bang D., Awano H., Tominaga J., Kolobov A.V., Fons P., Saito Y., Makino K., Nakano T., Hase M., Takagaki Y. (2014). Mirror-symmetric Magneto-optical Kerr Rotation using Visible Light in [(GeTe)_2_(Sb_2_Te_3_)_1_]*_n_* Topological Superlattices. Sci. Rep..

[92] Lotnyk A., Bernütz S., Sun X., Ross U., Ehrhardt M., Rauschenbach B. (2016). Real-space imaging of atomic arrangement and vacancy layers ordering in laser crystallised Ge_2_Sb_2_Te_5_ phase change thin films. Acta Mater..

[93] Bragaglia V., Arciprete F., Zhang W., Mio A.M., Zallo E., Perumal K., Giussani A., Cecchi S., Boschker J.E., Riechert H. (2016). Metal-Insulator Transition Driven by Vacancy Ordering in GeSbTe Phase Change Materials. Sci. Rep..

[94] Zhang B., Wang X.P., Shen Z.J., Li X.B., Wang C.S., Chen Y.J., Li J.X., Zhang J.X., Zhang Z., Zhang S.B. (2016). Vacancy Structures and Melting Behavior in Rock-Salt GeSbTe. Sci. Rep..

[95] Hilmi I., Lotnyk A., Gerlach J.W., Schumacher P., Rauschenbach B. (2017). Epitaxial formation of cubic and trigonal Ge-Sb-Te thin films with heterogeneous vacancy structures. Mater. Des..

[96] Park Y.J., Cho J.Y., Jeong M.W., Na S., Joo Y.C. (2016). New pathway for the formation of metallic cubic phase Ge-Sb-Te compounds induced by an electric current. Sci. Rep..

[97] Nam S.W., Chung H.S., Lo Y.C., Qi L., Li J., Lu Y., Johnson A.T., Jung Y., Nukala P., Agarwal R. (2012). Electrical wind force-driven and dislocation-templated amorphization in phase-change nanowires. Science.

[98] Nukala P., Agarwal R., Qian X., Jang M.H., Dhara S., Kumar K., Johnson A.T., Li J., Agarwal R. (2014). Direct observation of metal-insulator transition in single-crystalline germanium telluride nanowire memory devices prior to amorphization. Nano Lett..

[99] Pirovano A., Lacaita A.L., Pellizzer F., Kostylev S.A., Benvenuti A., Bez R. (2004). Low-field amorphous state resistance and threshold voltage drift in chalcogenide materials. IEEE Trans. Electron Devices.

[100] Ielmini D., Lacaita A.L., Mantegazza D. (2007). Recovery and Drift Dynamics of Resistance and Threshold Voltages in Phase-Change Memories. IEEE Trans. Electron Devices.

[101] Fantini P., Brazzelli S., Cazzini E., Mani A. (2012). Band gap widening with time induced by structural relaxation in amorphous Ge_2_Sb_2_Te_5_ films. Appl. Phys. Lett..

[102] Fantini P., Ferro M., Calderoni A., Brazzelli S. (2012). Disorder enhancement due to structural relaxation in amorphous Ge_2_Sb_2_Te_5_. Appl. Phys. Lett..

[103] Mitrofanov K.V., Kolobov A.V., Fons P., Wang X., Tominaga J., Tamenori Y., Uruga T., Ciocchini N., Ielmini D. (2014). Ge L3-edge X-ray absorption near-edge structure study of structural changes accompanying conductivity drift in the amorphous phase of Ge_2_Sb_2_Te_5_. J. Appl. Phys..

[104] Raty J.-Y., Zhang W., Luckas J., Chen C., Bichara C., Mazzarello R., Wuttig M. (2015). Aging mechanism of amorphous phase change materials. Nat. Commun..

[105] Polking M.J., Zheng H., Ramesh R., Alivisatos A.P. (2011). Controlled synthesis and size-dependent polarization domain structure of colloidal germanium telluride nanocrystals. J. Am. Chem. Soc..

[106] Giusca C.E., Stolojan V., Sloan J., Borrnert F., Shiozawa H., Sader K., Rummeli M.H., Buchner B., Silva S.R. (2013). Confined crystals of the smallest phase-change material. Nano Lett..

[107] Xu M., Cheng Y.Q., Wang L., Sheng H.W., Meng Y., Yang W.G., Han X.D., Ma E. (2012). Pressure tunes electrical resistivity by four orders of magnitude in amorphous Ge_2_Sb_2_Te_5_ phase-change memory alloy. Proc. Natl. Acad. Sci. USA.

[108] Caravati S., Bernasconi M., Kühne T., Krack M., Parrinello M. (2009). Unravelling the Mechanism of Pressure Induced Amorphization of Phase Change Materials. Phys. Rev. Lett..

[109] Caravati S., Sosso G.C., Bernasconi M., Parrinello M. (2013). Density functional simulations of hexagonal Ge_2_Sb_2_Te_5_ at high pressure. Phys. Rev. B.

[110] Krbal M., Kolobov A., Haines J., Fons P., Levelut C., Le Parc R., Hanfland M., Tominaga J., Pradel A., Ribes M. (2009). Initial Structure Memory of Pressure-Induced Changes in the Phase-Change Memory Alloy Ge_2_Sb_2_Te_5_. Phys. Rev. Lett..

[111] Sun Z., Zhou J., Pan Y., Song Z., Mao H.K., Ahuja R. (2011). Pressure-induced reversible amorphization and an amorphous-amorphous transition in Ge_2_Sb_2_Te_5_ phase-change memory material. Proc. Natl. Acad. Sci. USA.

[112] Kalikka J., Zhou X., Dilcher E., Wall S., Li J., Simpson R.E. (2016). Strain-engineered diffusive atomic switching in two-dimensional crystals. Nat. Commun..

[113] Zhou X.L., Kalikka J., Ji X.L., Wu L.C., Song Z.T., Simpson R.E. (2016). Phase-Change Memory Materials by Design: A Strain Engineering Approach. Adv. Mater..

[114] Privitera S.M.S., Mio A.M., Smecca E., Alberti A., Zhang W., Mazzarello R., Benke J., Persch C., La Via F., Rimini E. (2016). Structural and electronic transitions in Ge_2_Sb_2_Te_5_ induced by ion irradiation damage. Phys. Rev. B.

[115] Simpson R.E., Fons P., Kolobov A.V., Fukaya T., Krbal M., Yagi T., Tominaga J. (2011). Interfacial phase-change memory. Nat. Nanotechnol..

[116] Tominaga J., Kolobov A.V., Fons P., Nakano T., Murakami S. (2014). Ferroelectric Order Control of the Dirac-Semimetal Phase in GeTe-Sb_2_Te_3_ Superlattices. Adv. Mater. Interfaces.

[117] Loke D., Lee T.H., Wang W.J., Shi L.P., Zhao R., Yeo Y.C., Chong T.C., Elliott S.R. (2012). Breaking the speed limits of phase-change memory. Science.

[118] Zhu M., Xia M., Rao F., Li X., Wu L., Ji X., Lv S., Song Z., Feng S., Sun H. (2014). One order of magnitude faster phase change at reduced power in Ti-Sb-Te. Nat. Commun..

[119] Xia M., Zhu M., Wang Y., Song Z., Rao F., Wu L., Cheng Y., Song S. (2015). Ti–Sb–Te Alloy: A Candidate for Fast and Long-Life Phase-Change Memory. ACS Appl. Mater. Interface.

[120] Kolobov A.V., Krbal M., Fons P., Tominaga J., Uruga T. (2011). Distortion-triggered loss of long-range order in solids with bonding energy hierarchy. Nat. Chem..

[121] Mitrofanov K.V., Fons P., Makino K., Terashima R., Shimada T., Kolobov A.V., Tominaga J., Bragaglia V., Giussani A., Calarco R. (2016). Sub-nanometre resolution of atomic motion during electronic excitation in phase-change materials. Sci. Rep..

[122] Bragaglia V., Jenichen B., Giussani A., Perumal K., Riechert H., Calarco R. (2014). Structural change upon annealing of amorphous GeSbTe grown on Si(111). J. Appl. Phys..

[123] Wang R., Zhang W., Momand J., Ronneberger I., Boschker J.E., Mazzarello R., Kooi B.J., Riechert H., Wuttig M., Calarco R. (2017). Formation of resonant bonding during growth of ultrathin GeTe films. NPG Asia Mater..

[124] Momand J., Wang R., Boschker J.E., Verheijen M.A., Calarco R., Kooi B.J. (2017). Dynamic reconfiguration of van der Waals gaps within GeTe-Sb_2_Te_3_ based superlattices. Nanoscale.

[125] Breznay N.P., Volker H., Palevski A., Mazzarello R., Kapitulnik A., Wuttig M. (2012). Weak antilocalization and disorder-enhanced electron interactions in annealed films of the phase-change compound GeSb_2_Te_4_. Phys. Rev. B.

[126] Liao J., Ou Y., Feng X., Yang S., Lin C., Yang W., Wu K., He K., Ma X., Xue Q.K. (2015). Observation of Anderson localization in ultrathin films of three-dimensional topological insulators. Phys. Rev. Lett..

[127] Ying T.P., Gu Y.Q., Chen X., Wang X.B., Jin S.F., Zhao L.L., Zhang W., Chen X.L. (2016). Anderson localization of electrons in single crystals: Li_x_Fe_7_Se_8_. Sci. Adv..

